# CSF Markers of TG6 Autoimmunity in Gluten Ataxia

**DOI:** 10.1007/s12311-025-01832-z

**Published:** 2025-04-02

**Authors:** Mara-Luciana Floare, Stephen B. Wharton, Julie E. Simpson, Daniel Aeschlimann, Marios Hadjivassiliou

**Affiliations:** 1 Sheffield Institute for Translational Neuroscience, The University of Sheffield, Sheffield, UK; 2 Matrix Biology & Tissue Repair Research Unit, College of Biomedical and Life Sciences, Cardiff University, Cardiff, UK; 3 Academic Departments of Neurosciences and Neuroradiology, Sheffield Teaching Hospitals NHS Trust, Sheffield, UK

**Keywords:** Ataxia, Neuroinflammation, Plasma cells, Transglutaminase

## Abstract

Gluten ataxia (GA) is the primary neurological manifestation of gluten sensitivity, characterised by loss of Purkinje cells throughout the cerebellar cortex and rooted in autoimmunity to transglutaminase 6 (TG6). Previous studies have shown the contribution of serum anti-TG6 antibodies to disease progression; however, it remains unclear where these antibodies are produced and how they gain access into the brain parenchyma. This study aims to provide an immunological assessment of the CSF in patients with GA to better define the humoral response contributing to disease pathophysiology. In this observational study we assessed the presence of plasma cells in the CSF of 20 patients with GA and 6 controls. CSF from 16 of the GA patients and from all 6 controls was investigated for the presence of anti-TG6 IgA antibodies. Immunohistochemistry for CD138 was performed to assess the presence of plasma cells in the cerebellum and spinal cord of 4 cases with GA, 4 ataxia controls and 4 neurologically healthy controls. A significant increase in anti-TG6 IgA antibodies was detected in the CSF of patients with GA compared to controls, with no correlation between CSF and serum levels of anti-TG6 IgA antibodies for either experimental group. CD138^+^ cells were present in the CSF of 2 patients with GA and in the cerebellum and spinal cord of 3 post-mortem cases of GA. In a subpopulation of patients with GA intrathecal presence of plasma cells and TG6 antibodies is a feature of the disease, likely associated with prolonged disease duration and continuous exposure to gluten.

**Supplementary Information:**

The online version contains supplementary material available at 10.1007/s12311-025-01832-z.

## Introduction

Gluten sensitivity is a complex, autoimmune multisystem disease with diverse manifestations affecting the gastrointestinal (GI) tract, the central and peripheral nervous systems (CNS) [1] and the skin [2]. Amongst the neurological manifestations, cerebellar involvement, also known as gluten ataxia (GA), is the commonest [1]. Clinically, there are no unique features that distinguish GA from other forms of cerebellar ataxias and a positive diagnosis is made based on the presence of insidious (on rare occasions acute and rapidly progressive) onset of ataxia, with preferential involvement of the vermis resulting in gait ataxia [3] in the presence of circulating anti-gliadin antibodies indicative of gluten sensitivity [4].

Although mechanistic studies are rare, there is strong evidence to suggest that autoimmunity to transglutaminase 6 (TG6), a transglutaminase expressed in the brain, is part of GA pathophysiology and could contribute to the degeneration of cerebellar Purkinje cells observed on post-mortem examination [3, 5]. Passive transfer of serum from patients with GA positive for anti-gliadin and anti-TG6 antibodies caused severe ataxia in mice 3h and 6h post-injection and showed discrete cytoplasmic reactivity with Purkinje cells using immunohistological methods [6]. When serum antigliadin antibodies were immunodepleted with crude gliadin in this model, the immunoreactive profile persisted, indicating that Purkinje cells are a potential target of serum anti-TG6 antibodies [7]. Additionally, the pathological significance of anti-TG6 antibodies has been evidenced clinically, through the observation that adherence to a gluten-free diet (GFD) leads to a successful reduction in serum antibody titres and improvement of symptoms. However, despite evidence for a pathological involvement of serum TG6-antibodies in GA, it remains unclear where these antibodies are produced, and if and how they gain access into the brain parenchyma. Previous cerebrospinal fluid (CSF) case reports demonstrated the presence of anti-gliadin antibodies in one patient with coeliac disease (CD) and ataxia [8] and an upregulation in chemokine IP-10 and oligoclonal bands in patients with GA compared to controls [9], providing support for a humoral response in gluten ataxia. However, further characterisation of the CSF of patients with GA is lacking.

In the current study we hypothesise that immunoglobulin A (IgA) anti-TG6 antibodies produced in the gut gain access into the CNS through a dysfunctional blood-brain barrier (BBB) and contribute to cerebellar degeneration in GA patients. To test our hypothesis, we undertook an immunological analysis of CSF from patients with GA and controls to investigate the presence of plasma cells and IgA anti-TG6 antibodies. Additionally, immunohistochemical analysis of post-mortem (PM) material was performed to investigate the presence of plasma cells in the brain parenchyma.

## Materials and Methods

### Patient Selection

Paired serum and CSF samples were collected from patients attending the Sheffield Ataxia Clinic based at the Academic Neuroscience Department, Royal Hallamshire Hospital, Sheffield, United Kingdom. Informed consent was obtained from all patients and the study was approved by a Research Ethics Committee (IRAS ID 288752). Data were collected from 20 patients with GA (Supplementary Table 1) and 6 patients with headaches who underwent CSF examination representing the control group.

### Study Cohort for PM Analysis

PM human CNS tissue was obtained from the Sheffield Brain Tissue Bank, following ethical approval (REC19/SS/0029). Data were collected from the cerebellum and spinal cord of a total of 4 patients with gluten ataxia, 4 patients with other forms of ataxia (3 with multiple system atrophy confirmed at PM and one with genetically confirmed Friedreich’s Ataxia) who represented the ataxia disease control group and 4 neurologically healthy controls (Table 1). The clinicopathological findings in these cases have been reported in a separate study [10].

**Table 1:**
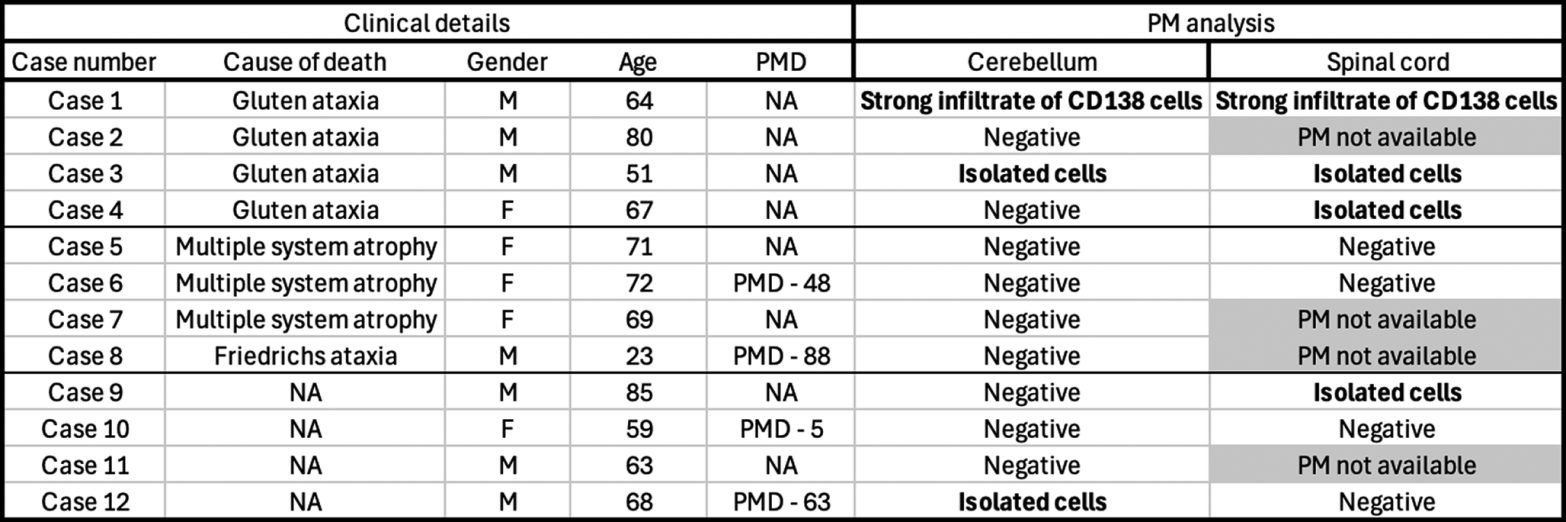
Demographic Table of post-mortem Cases and Histological Findings. PMD = post-mortem delay (in hours)

### CSF Immunological Analysis

#### H&E Staining and CD138 Immunocytochemical Analysis of Cytospin Preparations

Haematoxylin and eosin (H&E) and CD138 immunostains were performed on cytospin preparations by the Histopathology Laboratory at Sheffield Teaching Hospitals NHS Trust. All steps were carried out at RT. Briefly, 5 mL of CSF were centrifuged for 5 min at 500xg. The pellet was resuspended in 100μL supernatant and added into the cytospin centrifuge funnel. After centrifuging at 500xg for 3 min slides were stained for H&E on the MYREVA SS-30 Stainer (Especialidades Medicas Myr, S.L., Spain) using the built-in H&E programme. For CD138 immunocytochemistry, thymus sections were used as a positive control. All slides were placed in Dako Wash Buffer for 10 min and incubated with ready-to-use primary mouse anti-human CD138 antibody (Dako Omis, Allegiant) for 50 min. Slides were washed in Dako Wash Buffer for 5 min and incubated with EnVision FLEX Peroxidase-Blocking Reagent for 10 min. EnVision FLEX Mouse Linker was added for 10 min and the slides were washed for 5 min in Dako Wash Buffer before incubating for 30 min with Envision FLEX/HRP reagent.

The cytospin samples were visualised and imaged using a Nikon microscope (Nikon Instruments Inc, USA) and assessed blind to any clinical information by a consultant neuropathologist.

### Detection of TG6-IgA in Serum and CSF by ELISA

Detection of IgA anti-TG6 was performed using the commercially available TG6 IgA ELISA (Zedira, Germany) and all steps were performed according to the manufacturer’s instructions with minor modifications. All steps were performed at RT unless specified otherwise. Briefly, prior to use, each well of the solid phase was washed in 350µL wash buffer for 10s. All washing steps were performed using an automated washing machine. 300µL of undiluted patient CSF/100µl of serum (diluted 1/100 in sample buffer), controls and calibrators were dispensed into the microwells, all in duplicate. All binding steps were carried out for 30 min and were followed by four washes with 350µL wash buffer per well. Antibody binding was detected by incubating with anti-h-IgA HRP conjugate. The reaction was developed for 30 min using 100µL substrate solution in the dark and stopped with 100µL of stop solution. After 10s, the absorbance at 450 nm and 620 nm was read by a microplate photometer.

### Immunohistochemistry for CD138

Immunohistochemistry on paraffin-embedded formalin-fixed tissue sections for cluster of differentiation 138 (CD138) was performed by the Pathology Department at The Sheffield Teaching Hospital NHS Foundation Trust using Dako Autostainer. Ready-to-use anti-CD138 antibody (Dako) was incubated with the tissue sections for 20 min and the remaining steps were performed as indicated above for CD138 immunocytochemical analysis.

### Data Analysis

ELISA data was analysed using Prism (GraphPad Software InC.). A nonparametric Mann-Whitney test was performed to determine the variation in CSF IgA between the study groups. A Spearman rank correlation was performed to investigate the presence of any correlations between serum and CSF IgA anti-TG6 antibody levels.

Qualitative assessment of histological staining was performed using a Nikon microscope (Nikon Instruments Inc, USA).

### Data Availability Statement

All data relevant to the study are included in the article or uploaded as supplementary information.

## Results

### CD138 Positive Cells in CSF in GA

**Fig. 1:**
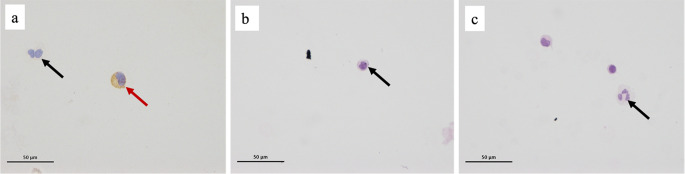
Analysis of CSF samples for the detection of plasma cells. Isolated CD138^+^ cells were observed on immunocytochemical staining from 2 GA patients (red arrow in **a**), together with monocytes (black arrow in **a**, **b**) and neutrophils (black arrow in **c**)

CD138^+^ cells were found in 2 out of 16 (12.5%) of GA cases and in no controls (Fig. 1A). The 2 patients with CD138 positive cells in their CSF had the longest duration of disease (one patient had CD for over a decade but was not adherent to the GFD and the other had a 20 year history of progressive ataxia before he was diagnosed with GA). Additionally, lymphocytes, monocytes and/or neutrophils were present in the CSF of 12 patients with GA (75%) and 6 controls (100%) (Fig. 1B, C) (Table 1).

### Anti-TG6 Antibodies Detected in CSF from Patients with GA

**Fig. 2:**
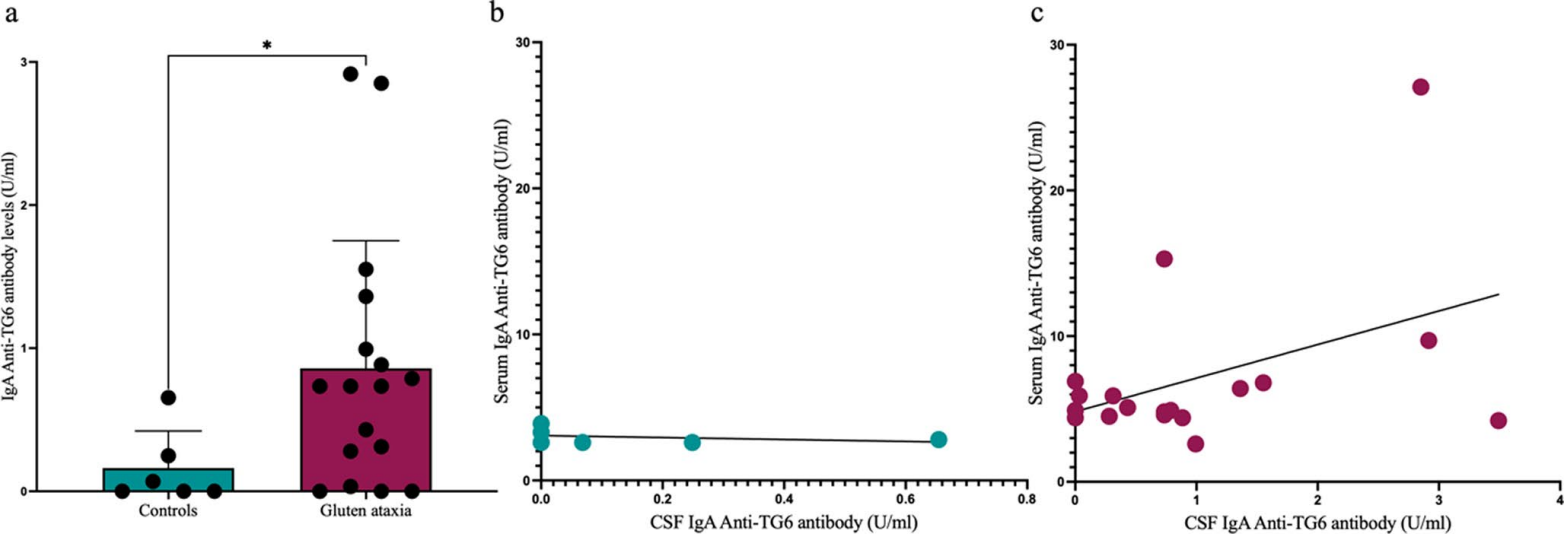
Detection of IgA anti-TG6 antibodies in CSF from GA cases and controls. Antibody titres in the GA group were significantly higher compared to the control group (*p* = 0. 0239). Values below 1U/mL were considered negative (**a**). There was no correlation between serum and CSF IgA anti-TG6 antibody levels in controls (*R*=-0.3548, *p* = 0.5250) (**b**) or in GA patients (*R* = 0.0954, *p* = 0.7063) (**c**)

Antibody titres were significantly higher in the GA group (median = 0.7347, IQR = 1.777-0.1570) compared to the control group (median = 0.0342, IQR = 0.3504-0), (*p* = 0.0239) (Fig. 2A). The CSF of the 2 cases in which CD138^+^ cells were identified measured a titre of 1.36 U/mL and 0.88 U/mL of anti-TG6 IgA.

### There is no Correlation Between Serum and CSF IgA Anti-TG6 Antibodies in Patients with GA

There was no correlation between serum and CSF IgA anti-TG6 antibody levels in GA (*R* = 0.1896, *p* = 0.4369)(Fig. 2C) nor in controls (*R*=-0.3548, *p* = 0.5250) (Fig. 2B).

### Plasma Cells Infiltration Is Present in the Cerebellum and Spinal Cord of Patients with GA

**Fig. 3:**
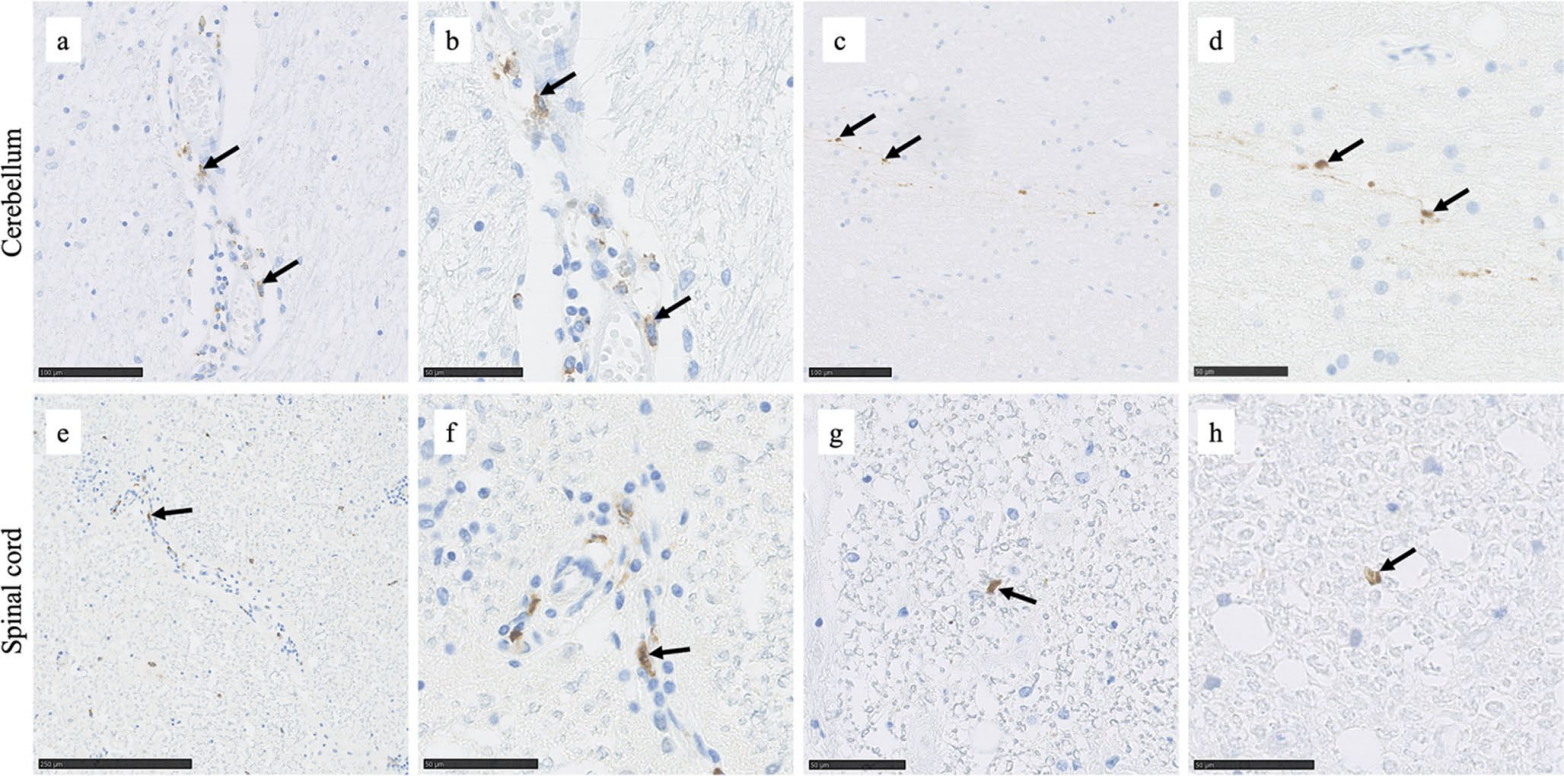
CD138^+^ lymphocytic infiltration in the cerebellum and spinal cord. CD138^+^ cells were present in a rich infiltrate in the cerebellum of GA case 1 (**a**, **b**) and to a lesser extend in GA case 3 (**c**. **d**). A rich infiltrate of positive cells was observed in the dorsal collum of GA case 1 (**e**, **f**), while isolated cells were present in GA case 3 (**g**) and case 4 (**h**)

A rich infiltrate of CD138^+^ cells was observed in the cerebellum (Fig. 3A, B) and dorsal column (Fig. 3E, F) of one case with GA. Additionally, isolated CD138^+^ cells were observed in the cerebellar white matter of one other GA case (out of 4 investigated) (Fig. 3C, D) and one healthy control and in the spinal cord of 2 two other GA cases (Fig. 3G, H) and one other healthy control case.

## Discussion

Cerebellar degeneration results in loss of gait balance, uncoordinated movements and slurred speech that characterise GA [11]. However, little is known about the molecular mechanisms that lead to death of cerebellar cells. Previous reports proposed a contribution of TG6 autoimmunity to GA pathogenesis [12] with a focus on serological assessments, but hardly any research is centred on investigating the CSF. In this study we performed an immunological characterisation of paired serum and CSF samples from patients with GA and controls and demonstrate the presence of CD138^+^ cells in the CSF of some but not all patients with GA and a significant increase in IgA anti-TG6 antibody titres in the CSF of patients with GA compared to controls. Additionally, we show the presence of CD138^+^ lymphocytic infiltrates in the cerebellum and spinal cord of 3 post-mortem cases of GA.

CD138 is a transmembrane protein associated with the plasma cell stages of B cell differentiation [13]. Under homeostatic conditions, CD138^+^ plasma cells are primarily found in lymphoid tissue where they contribute to effective immune surveillance and antibody production [14]. However, under pathological conditions, plasma cells can migrate to sites of infection and chronic inflammation, contributing to humoral immune responses and maintaining long-term immunity. The presence of B-cells and oligoclonal bands in the CSF of patients with multiple sclerosis (MS) has long been recognised as a key feature of the disease, providing evidence for an inflammatory process underlying its pathogenesis [15]. Additionally, recent reports found high levels of CD138 protein in the CSF of patients with MS at all disease stages, as well as CD138^+^ plasma cells being present in a perivascular location in PM material from one patient with relapsing remitting multiple sclerosis (RRMS), suggesting that CD138 could act as a specific CSF biomarker for MS [16]. Similarly, the finding of CD138^+^ cells in the CSF of two patients with GA and CD138 immunoreactivity in the spinal cord and perivascularly in the cerebellar white matter reported here provide further evidence for an underlying inflammatory process contributing to GA pathophysiology. It is important to note that the presence of CD138^+^ cells in the CSF was not observed across the whole cohort but only seen in patients with a long disease duration and no adherence to GFD at the time the CSF sample was collected: the first patient was diagnosed with CD 10 years before but was not adhering to the GFD and the second case had a history of slowly progressive ataxia for 20 years before being diagnosed with GA. Similarly, CD138^+^ infiltration was more prevalent in the PM case with the greater pathological burden [10]. This observation raises the question of whether the prolonged period of untreated disease could explain the more active CSF and the presence of CD138^+^ cells in these cases, however due to limited number of cases available we were unable to establish a definitive relationship to disease duration. Adherence to a strict GFD is the mainstay of treatment of GA but the use of immunosuppressive drugs could also be justified in the context of such a neuroinflammatory response. The optimal timing of immunosuppression remains unclear. We recently demonstrated high levels of major histocompatibility complex II (MHC-II)^+^ microglia in the cerebellum of 4 patients with GA. Similarly, higher levels of immunoreactivity and overall cerebellar atrophy were observed in the 2 patients that were not on the GFD, indicating that continuous exposure to gluten, could perpetuate the immune response and exacerbate neuronal death [10].

Since immunoglobulin G (IgG) is the most prevalent immunoglobulin in the CSF under homeostatic conditions [17], one would expect intrathecal CD138^+^ cells to be associated with an increase in IgG levels. However, whilst no IgG anti-TG6 antibodies were detected in our preliminary analysis (unpublished data), an increase in CSF TG6-IgA levels was detected in the GA group compared to controls, possibly indicating intrathecal antibody synthesis by infiltrating inflammatory cells or diffusion of antibodies from the periphery via a compromised BBB. These findings complement our previous research which demonstrated TG6 colocalization with perivascular IgA deposits in the cerebellum of a patient with GA [3]. If intrathecal antibody synthesis is to be the source of TG6-IgA in the CSF, this would imply local proliferation and maturation of B-lymphocytes into IgA-secreting TG6-plasma cells. Under the hapten-carrier model proposed for CD [18], proliferation of IgA-secreting TG2-plasma cells is constrained by the presence of a localised gluten-specific T cell response. Given that introduction of a GFD is associated with a reduction in serum anti-TG6 antibody titres and improvement of symptoms in patients with GA, it is possible that a similar gluten-specific T cell response is involved in the activation of TG6-B-cells in GA. However, if activation and proliferation of TG6-B cells is localised to the CNS, the existence of gliadin peptides at this site would also be required for the presence of gluten-specific T cells. Therefore, we suggest that TG6-B cell differentiation and maturation occurs outside of the CNS, either at the gut level or in tertiary lymphoid structures. However, it remains unclear whether the intrathecal TG6-IgA antibodies reported here are produced by TG6-plasma cells which once differentiated, migrate to the CNS to regulate neuroinflammatory responses, or whether they originate from gut-resident TG6-plasma cells and extravasate into the brain parenchyma via a dysfunctional BBB. Rojas et al. (2019) [19] previously reported the presence of gut derived IgA-producing plasma cells in the CNS of a murine model of experimental autoimmune encephalomyelitis. Additionally, Fitzpatrick et al. (2020) [20] indicated that IgA-secreting plasma cells accumulate in the mouse dura mater in health and disease, forming an immunological barrier that protects against the spread of pathogens into the CNS. These studies challenge the current understanding of neuroimmunity and provide evidence for plasma cell migration out of intestinal niches and up to higher cortical levels. On the other hand, unpublished work from our group indicates BBB breakdown in post-mortem of patients with GA, evidenced by the presence of plasma protein albumin and fibrinogen into the brain parenchyma. This is supported by previous work demonstrating IgA deposits in the cerebellum and brain stem of patients with GA, particularly within the muscular layer of blood vessels [21]. Calculating a CSF IgA index in our cases would be a reliable way to further investigate the origin of the TG6-IgA antibodies reported here, however due to lack of sufficient material and experimental setting this was not possible.

Interestingly, there was no correlation between the titres of serum versus CSF IgA anti-TG6 antibodies in our cohort. Similar findings were previously reported in CD, where proteomic analysis on serum IgA and gut plasma cells isolated from patient duodenal biopsies indicated that serum IgA antibodies are not produced by gut plasma cells, but by an equivalent population of plasma cells which originate from the same B cell clone and migrate to the bone marrow [22]. This is in line with our hypothesis that IgA-secreting plasma cells migrate out of the GI system and into the CNS in GA. Alternatively, if TG6-IgA antibodies are produced in the gut and enter the CNS via the BBB, then the levels of antibodies accessing the brain parenchyma and the CSF would be dependent on the degree of BBB damage and antibody-antigen interactions in the cerebellum, which may explain the lack of correlation with serum levels. In support of this, no association in levels between serum and CSF antibodies were previously reported in patients with neuropsychiatric manifestations of systemic lupus erythematosus [23].

There are limitations to this work. It is important to acknowledge that small volumes of CSF were used for the detection of IgA anti-TG6 antibodies and plasma cells, which may prevent forming a more comprehensive understanding of the inflammatory response. Additionally, although the same commercially available ELISA assay was used for the detection of IgA anti-TG6 antibodies in CSF and serum, this assay had been manufactured and optimised for the detection of serum anti-TG6-antibodies, which are present at higher titres in patients with GA. Although further optimization experiments were performed as part of this research, it is possible that CSF IgA-anti TG6 measures were affected by low assay sensitivity. Therefore, future studies implementing ultra-sensitive biomarker detection technologies like MSD or Simoa should be used to validate and consolidate our findings. Additionally, our study is limited by the PM material available for analysis, and the small number of positive cells present which prevented us from performing further quantification.

## Conclusion

In conclusion, our study demonstrates that intrathecal presence of plasma cells and TG6-IgA antibodies is a feature of a subpopulation of patients with GA, possibly associated with prolonged disease duration and continuous exposure to gluten. Although the immunological mechanisms behind TG6-IgA synthesis remain to be addressed by future research, we propose that they represent an important feature of humoral immunity in GA.

## Electronic Supplementary Material

Below is the link to the electronic supplementary material.

Supplementary Material 1

## Data Availability

No datasets were generated or analysed during the current study.

## Abbreviations

BBB: Blood-brain barrier
CD: Coeliac disease
CD138: Cluster of differentiation 138
CNS: Central nervous system
CSF: Cerebrospinal fluid
GA: Gluten ataxia
GFD: Gluten-free diet
GI: Gastrointestinal
H&E: Haematoxylin and eosin
IgA: Immunoglobulin A
MHC-II: Major histocompatibility complex II
MRI: Magnetic resonance imaging
MS: Multiple sclerosis
NAA/Cr: N-acetyl aspartate/creatine
OCBs: Oligoclonal bands
PM: Post-mortem
RRMS: Relapsing remitting multiple sclerosis
TG6: Transglutaminase 6
